# Infection-Induced Retrotransposon-Derived Noncoding RNAs Enhance Herpesviral Gene Expression via the NF-κB Pathway

**DOI:** 10.1371/journal.ppat.1005260

**Published:** 2015-11-19

**Authors:** John Karijolich, Emma Abernathy, Britt A. Glaunsinger

**Affiliations:** 1 Department of Plant and Microbial Biology, University of California, Berkeley, Berkeley, California, United States of America; 2 Department of Molecular and Cell Biology, University of California, Berkeley, Berkeley, California, United States of America; 3 Howard Hughes Medical Institute, University of California, Berkeley, Berkeley, California, United States of America; University of Pennsylvania Medical School, UNITED STATES

## Abstract

Short interspersed nuclear elements (SINEs) are highly abundant, RNA polymerase III-transcribed noncoding retrotransposons that are silenced in somatic cells but activated during certain stresses including viral infection. How these induced SINE RNAs impact the host-pathogen interaction is unknown. Here we reveal that during murine gammaherpesvirus 68 (MHV68) infection, rapidly induced SINE RNAs activate the antiviral NF-κB signaling pathway through both mitochondrial antiviral-signaling protein (MAVS)-dependent and independent mechanisms. However, SINE RNA-based signaling is hijacked by the virus to enhance viral gene expression and replication. B2 RNA expression stimulates IKKβ-dependent phosphorylation of the major viral lytic cycle transactivator protein RTA, thereby enhancing its activity and increasing progeny virion production. Collectively, these findings suggest that SINE RNAs participate in the innate pathogen response mechanism, but that herpesviruses have evolved to co-opt retrotransposon activation for viral benefit.

## Author Summary

Short interspersed nuclear elements (SINEs) are noncoding mobile genetic elements that are present at ~10^6^ copies per mammalian genome, roughly comprising 10% of mammalian genomic real estate. SINEs are typically transcriptionally silenced, though in some cases viral infection can promote their expression, yet to an unknown functional outcome. Thus, SINE elements represent the largest class of infection-inducible noncoding RNAs that are functionally uncharacterized. Here, we reveal that SINE RNAs play a critical role in the host-pathogen interaction in that they are required for efficient murine gammaherpesvirus 68 (MHV68) replication and gene expression. We demonstrate that SINE RNAs, both exogenously expressed and infection-induced, are robust activators of the IKKβ kinase, a key signaling molecule in the innate immune response. Activation of the IKKβ kinase by SINE RNA is mediated through both MAVS-dependent and independent mechanisms. Moreover, we demonstrate the activation of the IKKβ via SINE RNA is required to drive the phosphorylation of MHV68 RTA, the main viral transcriptional activator, which enhances its transcriptional activating property. Collectively, we reveal the first example of a role for SINE RNAs in the host-pathogen interaction and identify them as a key immune signaling molecule early during infection. Though SINE RNAs activate the innate immune response, MHV68 has co-opted SINE-mediate innate immune activation to enhance the viral lifecycle.

## Introduction

While only ~1.5% of mammalian genomes consist of protein coding sequence, upwards of 75% of the genome is transcribed [1, 2]. A considerable amount of this transcription generates stable non-protein-coding RNAs (ncRNAs) of potential biological relevance. Similarly, transcription from the genomes of many large double stranded (ds) DNA viruses is pervasive and can generate an abundance of long and short ncRNAs, a number of which have key roles in viral replication and pathogenesis [3–11]. While there is an increasing appreciation that viruses have adopted ncRNAs as part of their gene regulatory repertoire, with the exception of some small ncRNAs such as microRNAs, how most other cellular ncRNAs may impact the gene expression landscape during infection remains unknown. Given that viruses have provided significant insight into mammalian gene regulation, they have the potential to reveal new features of ncRNA biology.

One of the largest potential sources of host-derived ncRNAs is a class of retrotransposons called short interspersed nuclear elements (SINEs), as these comprise greater than 10% of the human and mouse genomes [12–15]. SINEs are non-autonomous and require co-expression of protein products encoded within long interspersed nuclear elements (LINEs) for retrotransposition [16]. Alu elements are the predominant SINE family in humans, while the B1 and B2 SINEs are the major families in the murine genome. All SINE families are evolutionarily derived from endogenous RNA Polymerase III (Pol III) transcripts: Alu and B1 SINEs are derived from 7SL RNA, the RNA component of signal recognition particle, and B2 SINEs are derived from transfer RNA (tRNA) [17–20]. SINE elements contain internal Box A and Box B RNA Pol III promoter elements that drive transcription of a SINE ncRNA. In general, SINE elements are transcriptionally silenced in healthy somatic cells, although they can be activated by a variety of chemical and biological stresses [21]. In this regard, several viruses including herpes simplex virus 1 (HSV-1) [22, 23], adenovirus type 5 (Ad5) [24], and Minute virus of mice (MVM) have been shown to induce SINE RNA expression upon infection [25]. SINE elements are thus a robust source of inducible ncRNAs, whose expression could impact the gene expression environment during infection. Indeed, there is precedence for SINE RNA functioning in the regulation of gene expression during heat shock, where transcribed Alu and B2 SINE RNAs participate in transcriptional repression through direct interactions with RNA pol II [26–30].

Additional observations suggest that SINE ncRNA expression can also interface with components of the innate immune system, perhaps in a manner linked to their secondary structure. SINE RNAs are highly structured with multiple regions of long double stranded RNA (dsRNA) [31, 32], and the majority possess 5’-triphosphate moieties [33]. These ncRNAs thus have the potential to be recognized by cellular dsRNA sensors and could therefore serve as inducible immune signaling molecules. Early studies revealed that Alu RNA is efficiently bound by the double stranded RNA activated protein kinase (PKR) [34], and can function in either an inhibitory or activating capacity depending on the ncRNA concentration [35]. Furthermore, aberrant expression of Alu RNAs within retinal pigmented epithelium induces TLR-independent activation of the NLRP3 inflammasome, leading to geographic atrophy, a form of age-related macular degeneration (AMD) [36–38]. Thus, it is possible that mammalian cells have incorporated the regulated induction of SINE ncRNAs as a means to help control immune activation and gene expression, although aberrant or sustained SINE transcription is likely detrimental.

Here, we explored potential roles for SINE ncRNAs induced during viral infection using the murine gammaherpesvirus MHV68, which we found induces SINE ncRNA transcription in a rapid and sustained manner. MHV68 is a widely used model system for probing the *in vivo* biology and replication of gammaherpesviruses, a subfamily of large, nuclear replicating dsDNA viruses that includes the oncogenic human viruses Kaposi’s sarcoma-associated herpesvirus (KSHV) and Epstein-Barr virus (EBV). Unexpectedly, the induction of SINE ncRNAs boosts viral replication and gene expression, suggesting that herpesviruses have co-opted SINEs for proviral functions. The stimulatory effect on viral replication is linked to SINE RNA-based activation of the innate immune system, in particular the IKKβ component of the NF-κB signaling pathway. IKKβ is a known activator of the primary viral transcriptional transactivator protein RTA [39], and SINE expression enhances the IKKβ-dependent phosphorylation of RTA, thereby boosting its activity. We find that SINE RNAs activate NF-κB through both mitochondrial antiviral-signaling (MAVS) protein dependent and independent mechanisms. Collectively, our findings reveal that virus-induced retrotransposon expression contributes to activation of innate immune signaling during infection, but that herpesviruses exploit this pathway to bolster viral gene expression.

## Results

### MHV68 infection activates SINE transcription

**Fig 1:**
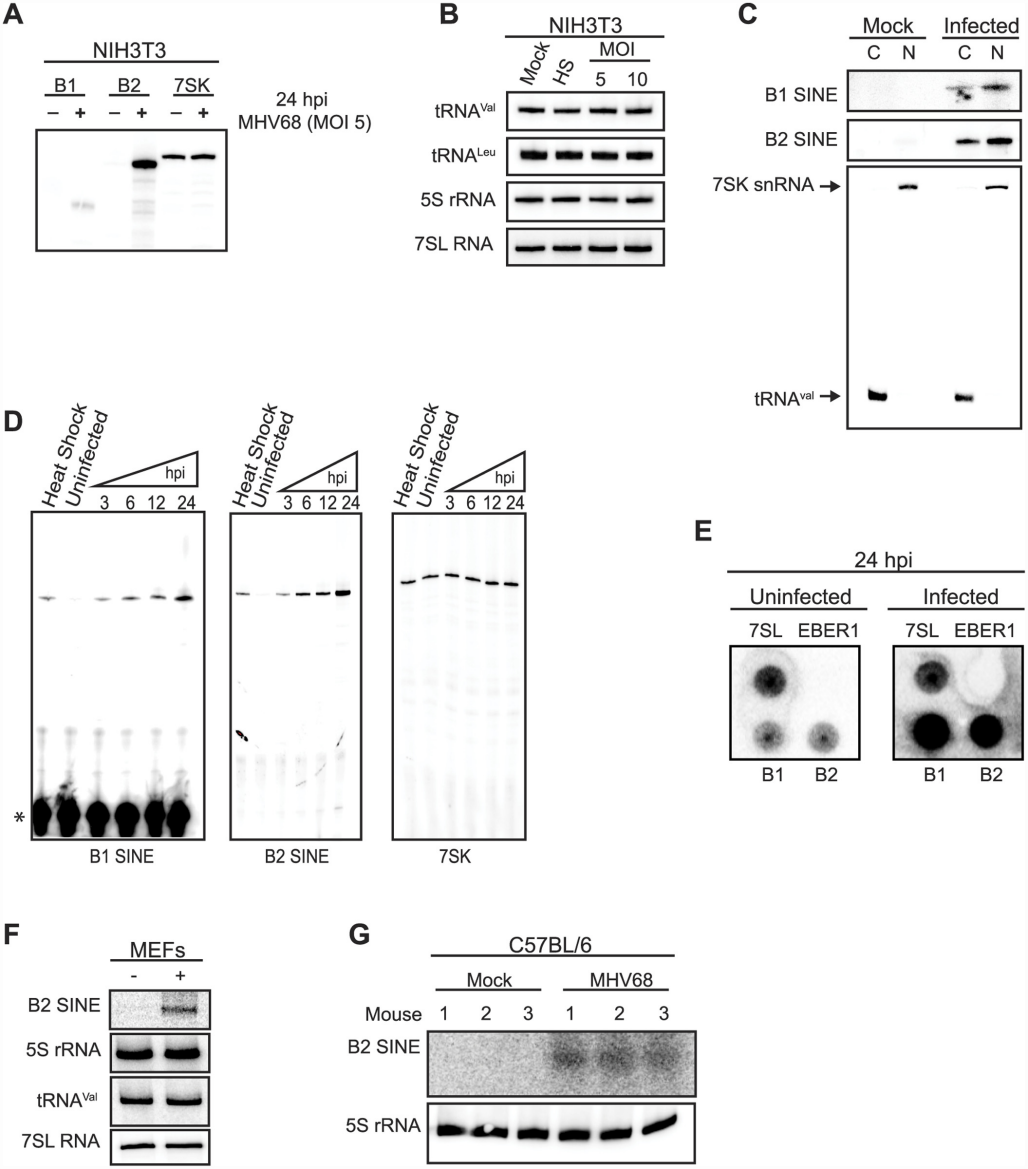
MHV68 infection induces SINE RNA expression with rapid kinetics. (**A**) NIH3T3 cells were infected with MHV68 at an MOI of 5, and 24 hpi total RNA was primer extended for B1, B2, and 7SK RNAs. (**B**) The RNA described in (A) was used to monitor the levels of tRNA^Val^, tRNA^Leu^, 5S rRNA, and 7SL RNA by small RNA northern blotting. Heat shock (HS) was used as an additional control. (**C**) RNA isolated from subcellular compartments of NIH3T3 cells infected with MHV68 for 24 h were primer extended (B1 and B2 RNAs) or northern blotted (7SK snRNA and tRNA^Val^). (C, cytoplasm; N, nucleoplasm.) (**D**) NIH3T3s were infected with MHV68 at an MOI 5 and total RNA was isolated at the indicated time points. The levels of B1 RNA, B2 RNA, and 7SK snRNA were monitored by primer extension. * denotes the unused radiolabed primer left in the gel. (**E**) Nuclei were isolated form NIH3T3s 24 hpi and used for nuclear run on analysis of 7SL RNA, EBER1, and B2 SINE. (**F**) MEFs were infected with MHV68 an MOI of 5 and 24 hpi total RNA was isolated and the levels of various RNA Pol III RNAs were determined by northern blotting. (**G**) C57BL/6 mice were intranasally infected with MHV68. 5 dpi lungs were excised and total RNA was isolated. The levels of B2 SINE RNA and 5S rRNA were monitored by northern blotting.

In unstressed somatic cells, SINE loci are transcriptionally repressed and thus RNA Pol III-transcribed SINE RNAs are either undetectable or only weakly expressed. To determine whether gammaherpesvirus infection activated SINE RNA expression, NIH3T3 cells were infected with MHV68 and B1 and B2 SINE RNA levels were quantified by primer extension. Indeed, both B1 and B2 SINEs were induced specifically upon infection, with B2 RNA levels exceeding those of the highly abundant RNA Pol III-transcribed 7SK small nuclear RNA (snRNA) (Fig 1A). This increase in RNA Pol III transcriptional activity during MHV68 infection was specific for SINE loci, as infected cells displayed no alteration in the levels of multiple other RNA Pol III-derived transcripts, including tRNA^Val^, 5S ribosomal RNA (rRNA), and 7SL RNA as measured by Northern blotting (Fig 1B). Additionally, B1 and B2 SINE RNAs localized to both the cytoplasmic and nuclear compartments (Fig 1C).

SINE RNA induction was both rapid and sustained, occurring by 3 h post infection (hpi) at levels similar to heat shock-driven induction and continually increasing throughout the 24 hpi time course (Fig 1D). During the same time course 7SK levels remained constant in both infected and uninfected cells (Fig 1D). To determine whether the increase in SINE RNAs occurred at the level of transcription, nuclear run-on assays were performed using nuclei from uninfected or MHV68-infected cells. A robust transcriptional increase was observed for both B1 and B2 in infected cells, whereas no transcriptional difference was observed for the RNA Pol III transcribed 7SL RNA (Fig 1E). No signal was detected for the negative control ncRNA EBER1 from Epstein-Barr virus, which is not expressed in these cells, confirming the specificity of the run-on signals (Fig 1E). Finally, the increase in SINE RNA was also observed upon infection of primary mouse embryonic fibroblasts (MEFs) and *in vivo* in the lung tissue of MHV68-infected C57BL/6 mice at 5 days post infection, indicating the induction was not restricted to established cell lines (Fig 1F and 1G). Together, these data demonstrate that MHV68 infection results in a rapid, sustained, and specific transcriptional activation of B1 and B2 SINE loci.

### Viral early gene expression is required for sustained SINE induction

**Fig 2:**
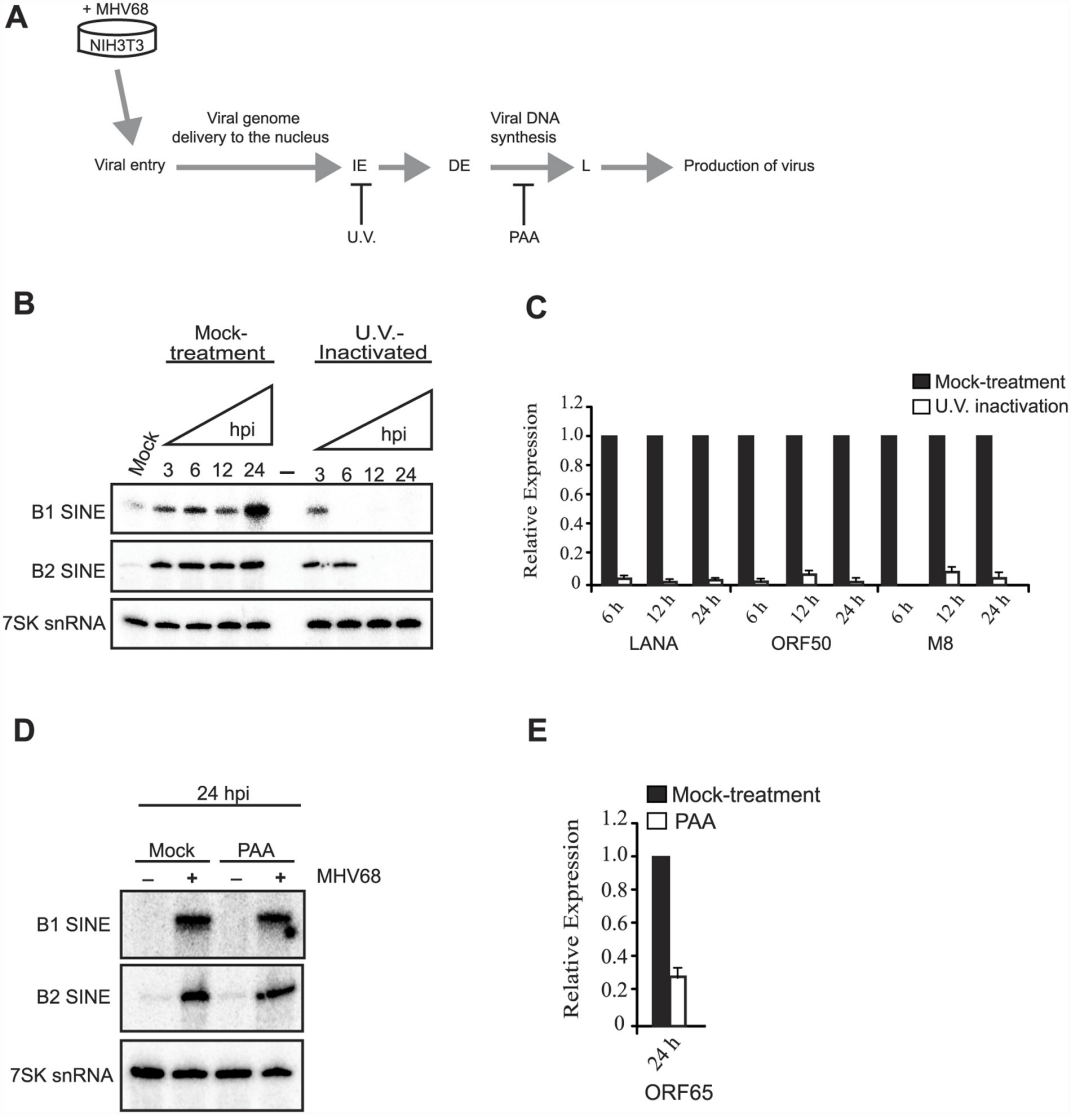
Viral gene expression is required for sustained SINE expression. **(A)** Temporal progression of the MHV68 productive replication cycle. Upon MHV68 infection, the virus enters cells and the dsDNA viral genome is delivered to the nucleus. The viral gene expression program commences with transcription of immediate early (IE) genes, which in turn activate transcription of early (E) genes. This is followed by viral DNA synthesis, which activates transcription of late (L) viral genes, and culminates in infectious progeny virus production. The point at which different treatments (UV-irradiated virus, or PAA) arrest the viral life cycle is shown. (**B**) RNA isolated from NIH3T3 cells incubated with mock- or U.V.-treated MHV68 at the indicated time points was subjected to RT-qPCR for the indicated viral genes to demonstrate that UV treatment prevents viral gene expression. (**C**) RNA described in (B) was used to monitor the levels of B1, B2, and 7SK RNA by primer extension. (**D**) NIH3T3 cells were infected with MHV68 at an MOI of 5 in the presence of 200 μg/mL of PAA. 24 hpi total RNA was used to monitor by RT-qPCR the expression of ORF65, a viral late gene whose transcription is dependent on viral DNA replication. (**E**) RNA described in (D) was used to monitor the levels of B1, B2, and 7SK RNA by primer extension.

We next sought to identify aspect(s) of the MHV68 life cycle responsible for SINE RNA induction. After entry, the viral genome is delivered to the nucleus and transcription of immediate early and early genes occurs, which in turn enable viral DNA replication and subsequent expression of late viral genes necessary for progeny virion assembly. UV crosslinking of viral particles does not block viral attachment to cells, but inactivates the viral genome thereby inhibiting viral gene expression and replication (Fig 2A and 2B). NIH3T3 cells incubated with UV-inactivated virus still displayed the early SINE RNA induction at 3 hpi, but unlike incubation with infectious virus, B1 and B2 RNA levels rapidly subsided over the 24 hpi time course (Fig 2C). (It should be noted that the B1 gels in Fig 2 were exposed longer than the B2 gels to better visualize the faint B1 signal). This suggests that viral activation of SINEs is biphasic, with the initial induction occurring in response to viral attachment and/or entry, and sustained SINE transcription reliant on downstream aspects of the viral life cycle.

To determine whether sustained SINE activation required viral genome replication or late gene expression, cells were treated with the viral DNA polymerase inhibitor phosphonoacetic acid (PAA) prior to MHV68 infection (Fig 2D). SINE RNA induction was similar at 24 hpi in control- and PAA-treated cells (Fig 2E). These data suggest that viral early gene expression, but not DNA replication or late gene expression drives sustained transcriptional activation of SINE loci. Although it is possible that a specific viral gene product might be responsible for SINE activation, we have been unable to identify any MHV68 genes whose individual expression in NIH3T3 cells induced SINE transcription. Thus, SINE RNA induction is likely to occur as a consequence of the combined activity of multiple viral genes and/or as a more general cellular response to infection.

### B2 SINE RNA enhances viral replication and gene expression

**Fig 3:**
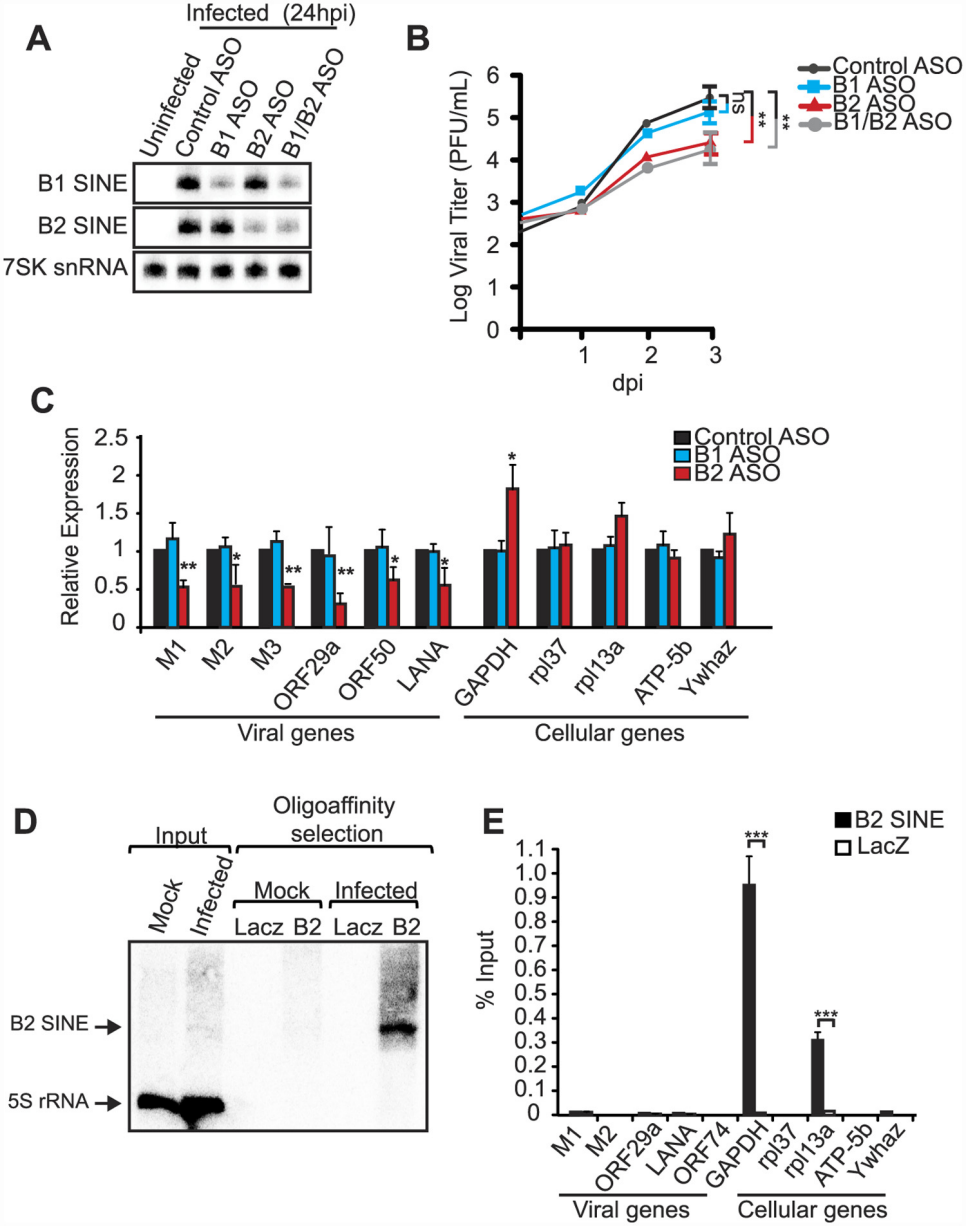
B2 RNA enhances viral replication and gene expression. (**A**) NIH3T3 cells transfected with indicated ASOs were infected with MHV68 at an MOI of 0.05. Infection was allowed to progress for 72 h. Primer extension was performed at 24 hpi infection to monitor the levels of B1, B2, and 7SK RNA. (**B**) Infectious virus produced in (A) was quantified by plaque assay in NIH3T3 cells. (**C**) RNA isolated at 72 hpi was used to monitor the levels of viral and cellular RNAs by RT-qPCR. (**D**) ChIRP was performed on NIH3T3 cells infected with MHV68 for 24 h. Antisense LacZ oligos were used as a negative control. RNAs isolated from the ChIRP experiment were analyzed by small RNA northern blotting for B2 SINE and 5S rRNA. (**E**) DNA isolated from the ChIRP experiment described in (D) was used for qPCR.

We hypothesized that the virus-induced noncoding SINE RNAs may play regulatory roles related to infection. To test whether SINE transcription impacts the MHV68 lifecycle, we measured viral replication upon specific knockdown of B1 or B2 SINE RNAs using 2′-*O*-methylated and phosphorothioate-substituted antisense oligonucleotides (ASO), which direct RNase H-based cleavage of target RNAs. Transfection of B1 and/or B2 ASOs 3 h prior to MHV68 infection of NIH3T3 cells significantly reduced the levels of SINE RNA at 24 hpi (Fig 3A). Control ASOs did not impact B1 or B2 levels, and ASO treatment of uninfected cells did not induce SINE expression (Fig 3A). Remarkably, B2 depletion resulted in delayed viral replication kinetics in a multistep growth curve and a ~15-fold decrease in progeny virion production (Fig 3B). We observed a similar (~10-fold) decrease in viral replication in a single-step growth curve upon treatment of NIH3T3 cells with the specific RNA Pol III inhibitor ML-60218 6 h prior to infection to block SINE transcription (S1 Fig). Although ML-60218 blocks transcription of all RNA Pol III genes, constitutive Pol III transcripts in general have long half-lives and thus, unlike B1 and B2 RNAs, their steady state levels are not appreciably affected by the treatment. Specific ASO-mediated depletion of B1 RNA, which accumulates to far lower levels than B2 RNA during MHV68 infection, had no effect on viral replication, and co-depletion of both B1 and B2 did not enhance the replication defect observed upon B2 knockdown (Fig 3B).

The above results indicated that induction of B2 SINE RNA by MHV68 is beneficial to the viral lifecycle. We therefore examined whether the SINE RNAs impacted viral gene expression by measuring the accumulation of viral mRNAs from different kinetic classes in cells treated with control or SINE-specific ASOs. Indeed, RT-qPCR analyses revealed that depletion of B2 RNA resulted in a significant reduction in the levels of all viral RNAs tested (Fig 3C). In agreement with the viral replication data, depletion of B1 RNA had no effect on viral gene expression. Interestingly, the effect of B2 RNA was primarily directed at viral genes, as we observed no decrease in the abundance of a set of 5 cellular mRNAs upon treatment with B1 or B2 ASOs (Fig 3C). However, we did note an increase in the levels of the GAPDH mRNA upon B2 depletion, in agreement with data showing that heat shock-induced B2 RNA can transcriptionally repress select promoters [26, 27]. We obtained similar results when SINE RNA transcription was inhibited by pre-treatment with ML-60218 (S1 Fig). Thus, virus-induced expression of B2 SINE RNA broadly enhances MHV68 mRNA abundance, and this stimulatory effect primarily impacts viral gene expression even though RNA Pol II transcribes both viral and cellular mRNAs.

### B2 SINEs are not associated with viral promoters

Several long noncoding RNAs manipulate gene expression through their recruitment to specific cellular gene promoters [40]. In this regard, B2 SINE RNA has been shown to interact with RNA Pol II at select promoters to inhibit transcription [26, 27]. Although this explains the apparent suppression of GAPDH mRNA by B2, the stimulatory effect of B2 on MHV68 mRNA suggested that SINE RNAs might not function through their recruitment to promoters. Using chromatin isolation by RNA purification (ChIRP) we examined whether B2 RNA was present at viral or cellular promoters. ChIRP-qPCR analyses detected B2 RNA at the promoter of GAPDH and RPL13a (Fig 3D and 3E). In contrast to our findings with cellular promoters, we were unable to ChIRP B2 RNA to any viral promoters, suggesting that B2 SINE RNA modulates viral gene expression indirectly.

### SINE RNAs activate the NF-κB signaling pathway

**Fig 4:**
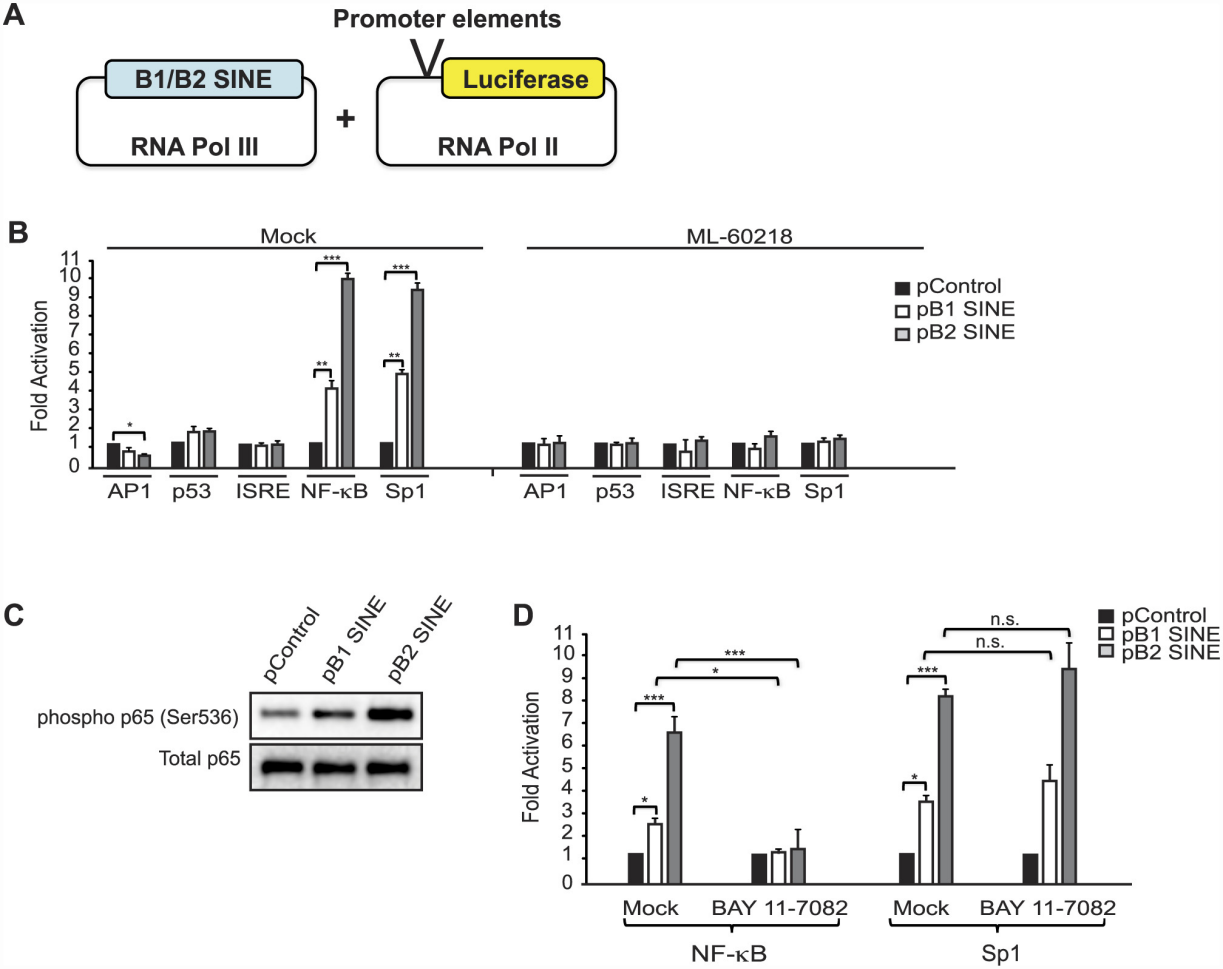
SINE RNAs activate the NF-κB pathway. (**A**) Schematic of the experimental design. B1 and B2 SINE expression constructs were co-transfected with reporter plasmids containing various promoter elements cloned upstream of the luciferase gene. (**B**) The indicated plasmids were co-transfected in to NIH3T3 cells and 48 h later luciferase levels were determined. (**C**) NIH3T3 cells were transfected with B1 or B2 expression constructs, or a control construct. 48 h later protein extracts were analyzed by western blot analysis. (D) The indicated plasmids were co-transfected in to NIH3T3 cells and 48 h later luciferase levels were determined.

Our above data suggested that SINE RNAs have promoter-specific effects on cellular genes. We tested this hypothesis by analyzing the effect of B1 and B2 SINE RNA expression on a panel of promoter elements cloned upstream of a luciferase reporter (Fig 4A). To monitor effects specifically linked to SINE RNAs as opposed to secondary effects stemming from infection, we co-expressed each reporter with consensus B1 or B2 SINEs derived from RNA Pol III-driven SINE expression constructs in NIH3T3 cells. SINE RNAs modestly suppressed the AP1 promoter, had no impact on the p53 and ISRE promoters, but significantly activated both the NF-κB-driven and Sp1-driven luciferase reporters (Fig 4B). In each case, B2 RNA had a more potent effect than B1, and all changes in luciferase reporter levels required SINE RNA expression, as they were blocked upon treatment with the RNA Pol III inhibitor ML-60218 (Fig 4B).

MHV68 infection has been shown to activate the NF-κB pathway, components of which are used by the virus for robust induction of lytic gene expression [39, 41–43]. Though MHV68 blunts the NF-κB transcriptional response by subsequently inducing degradation of the RelA/p65 subunit of NF-κB during the first 4 hpi [41], the IKKβ kinase component of the pathway is co-opted to promote phosphorylation of the major viral lytic transactivator RTA. Our findings suggested that induction of SINE RNAs might contribute to NF-κB activation during infection, thereby potentiating viral replication. To test this hypothesis, we first examined whether SINE RNA expression was sufficient to activate endogenous components of the NF-κB signaling pathway. Indeed, transfection of plasmid-based SINEs in uninfected cells resulted in increased phosphorylation of the NF-κB p65 subunit, a marker of NF-κB activation (Fig 4C). Furthermore, inhibition of IKKβ by treatment with its specific inhibitor BAY 11–7082 significantly reduced the ability of SINE RNA to activate the NF-κB luciferase reporter, but did not impact SINE-driven activation of the Sp1 reporter (Fig 4D). Thus, SINE RNAs likely activate this pathway in the cytoplasm, upstream of IKKβ.

### SINE RNAs signal through MAVS to activate IKKβ

**Fig 5:**
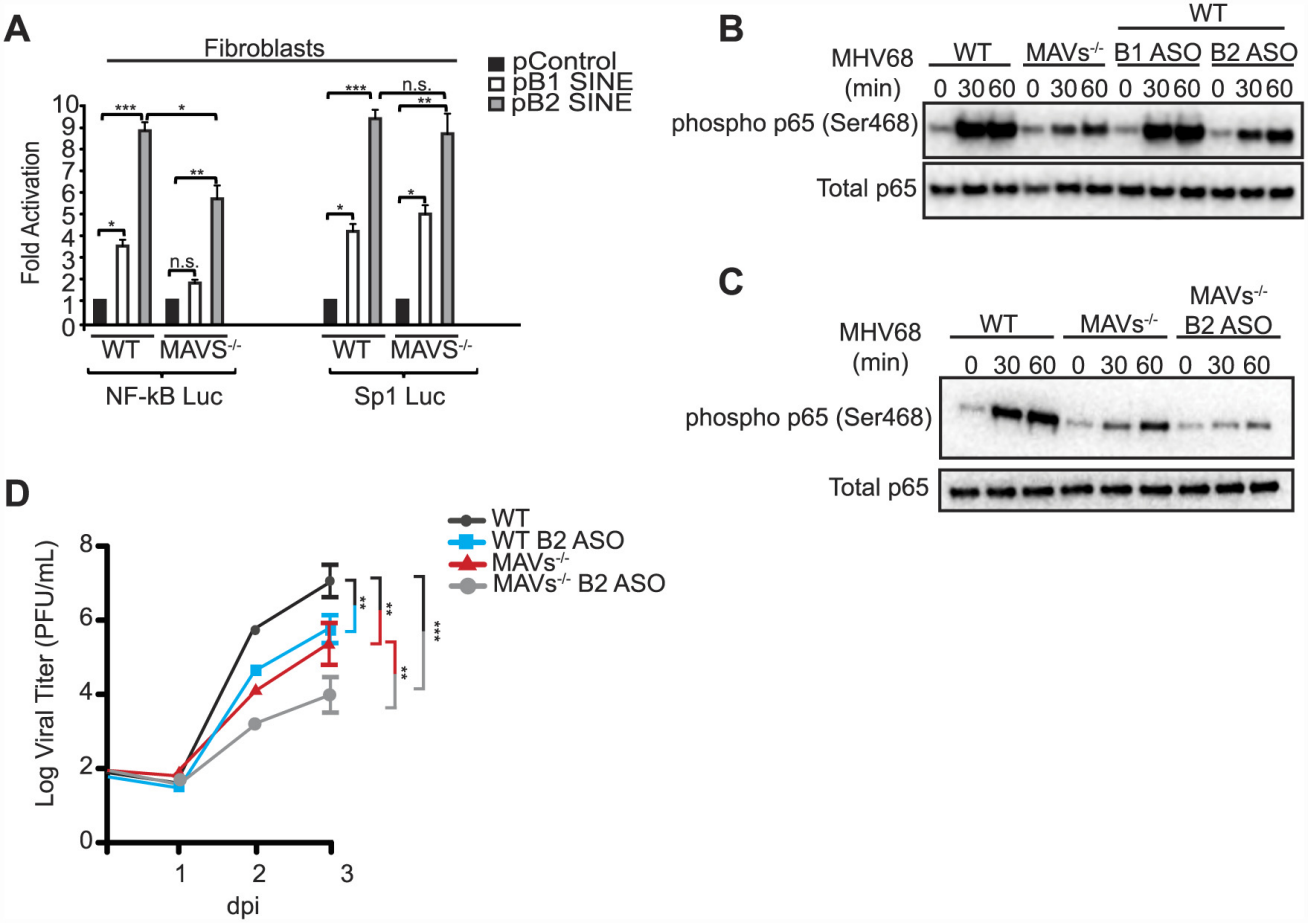
SINE RNAs signal through MAVS to activate NF-κB. (**A**) Control, B1 SINE, or B2 SINE expression constructs were co-transfected with either the NF-κB or Sp1 reporter luciferase plasmid into WT and MAVS^-/-^ fibroblasts. 48 h later luciferase levels were determined. (**B**) MAVS^-/-^, WT, and WT fibroblasts transfected with either B1 or B2 ASOs were infected with MHV68. At the indicated time points whole cell lysates were prepared and western blotted for phospho p65 (Ser468) and total p65. (**C**) WT, MAVS^-/-^, and MAVS^-/-^ fibroblasts transfected with B2 ASO were infected with MHV68 and western blot analysis was performed at the indicated times for phospho p65 (Ser468) and total p65. (**D**) WT and MAVS^-/-^ fibroblasts that are transfected with either control of B2 ASOs infected with MHV68 at an MOI of 0.05. Infection was allowed to progress for 72 h. Infectious virus produced was quantified by plaque assay in NIH3T3 cells.

IKKβ activation during MHV68 infection is at least partially mediated through activation of the MAVS adaptor protein [39]. However, the mechanism underlying MAVS activation during MHV68 infection remains unclear. MHV68 is a dsDNA virus, yet MAVS activation generally occurs upon recognition by upstream RIG-I-like receptors of nucleic acid features associated with RNA viruses, including double-stranded RNA with either a 5′ diphosphate or 5′ triphosphate [44]. We therefore explored whether the structured SINE RNAs might serve as the MAVS activation signal to stimulate IKKβ during MHV68 infection. Indeed, the ability of B1 or B2 SINE RNA expression to activate the NF-κB promoter was significantly, though not completely, impaired in MAVS^-/-^ fibroblasts relative to WT fibroblasts (Fig 5A). This effect was specific for the NF-κB promoter, as no decrease in B1- or B2-induced Sp1 promoter activation occurred in the cells lacking MAVS (Fig 5A).

We next examined the role of MAVS in endogenous, MHV68-induced SINE RNA-mediated phosphorylation of RelA/p65. Within 1 hpi, RelA/p65 is phosphorylated at Serine 468 in a MAVS-IKKβ-dependent manner, a mark that primes its degradation via the proteasome, thereby blunting the NF-κB response [41]. The ability of MHV68 to induce phosphorylation of RelA/p65 was significantly reduced in both MAVS^-/-^ fibroblasts and in WT fibroblasts that had been depleted of B2 RNA using a B2-specific ASO (Fig 5B). As anticipated, no effect was observed upon depletion of the B1 RNA, in agreement with the fact that it is only weakly induced relative to B2 RNA by MHV68 (Fig 5B). Additionally, MHV68-induced RelA/p65 phosphorylation was further reduced to background levels when B2 RNA was depleted in the MAVS^-/-^ cells, suggesting that at least a portion of SINE RNA-based IKKβ activation occurs in a MAVS-independent manner (Fig 5C). These results indicate that B2 RNA contributes to the blunting of the NF-κB response during MHV68 infection.

We next compared the relative importance of MAVS and B2 SINE RNA in productive MHV68 infection. MHV68 replication was impaired to a similar extent in fibroblasts lacking MAVS as in cells depleted of B2 SINE RNA, as measured in multi-step growth curves (Fig 5D). However, depletion of B2 RNA in a MAVS-/- background caused a further reduction in viral replication, to levels ~30-fold lower than in untreated WT cells. Collectively, these data indicate that the B2 RNAs are major contributors to the IKKβ activation observed during MHV68 infection, and that they stimulate this pathway via both MAVS-dependent and -independent mechanisms to boost viral replication.

### SINE RNAs enhance MHV68 RTA activity via IKKβ-mediated phosphorylation to increase viral gene expression

The broad enhancement of MHV68 gene expression by B2 RNA, coupled with the absence of B2 at viral promoters, suggested that SINE RNAs act at an early stage of the viral lifecycle to boost the subsequent gene expression cascade. Notably, the major viral lytic transactivator protein RTA was recently shown to be phosphorylated by IKKβ in a manner that increased RTA activity [39]. RTA is a viral protein expressed with immediate early kinetics, and its activity broadly impacts the viral gene expression cascade [45–47]. We therefore hypothesized that the pro-viral effects of the SINE RNAs might be mediated through an increase in RTA activity caused by B2-induced NF-κB pathway activation.

**Fig 6:**
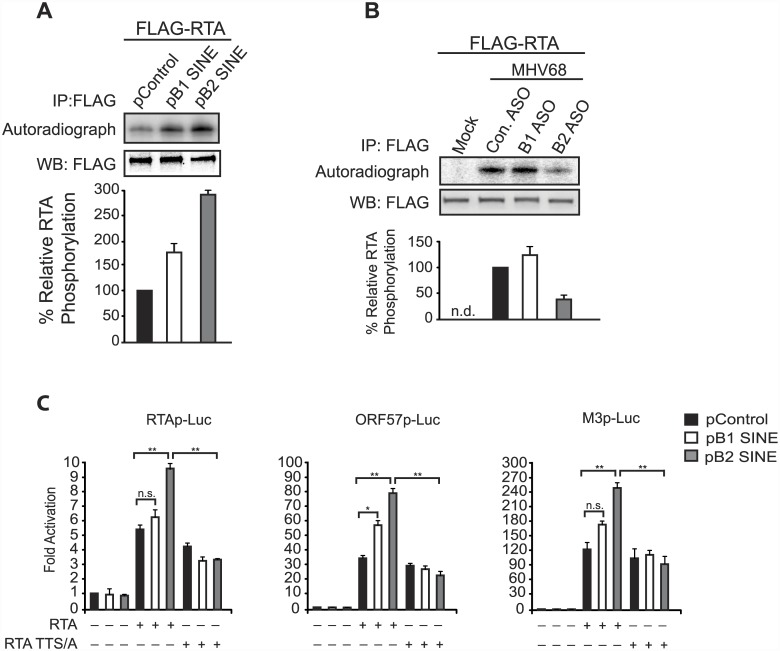
SINE RNA promotes IKKβ-mediated phosphorylation of RTA to potentiate viral transcription. (**A**) NIH3T3 cells were transfected with FLAG-RTA and either a control construct, B1 SINE, or B2 SINE expression plasmid. 24 h later cells were labeled with [^32^P]-orthophosphoric acid for 6 h. Whole cell lysates were precipitated with anti-FLAG antibody and analyzed by autoradiography (top) or western blot using an anti-FLAG antibody (bottom). (**B**) FLAG-RTA was transfected in to NIH3T3 cells. 24 h later cells were transfected with indicated ASOs and subsequently infected with MHV68 for 6 h in the presence of [^32^P]-orthophosphoric acid. Whole cell lysates were precipitated with anti-FLAG antibody and analyzed by autoradiography (top) or western blot using an anti-FLAG antibody (bottom). (**C**) NIH3T3 cells transfected with the indicated viral promoter luciferase plasmids were cotransfected with wild-type RTA or RTA TTS/A, and control, B1 SINE, or B2 SINE expression constructs. 48 h later luciferase levels were monitored.

We tested this idea first by examining whether the activation of IKKβ by B1 and B2 RNA could potentiate RTA phosphorylation, as measured by *in vivo* P^32^ orthophosphate labeling. FLAG-RTA was expressed in NIH3T3 cells in the presence or absence of B1 or B2 expression plasmids in ^32^P orthophosphate containing growth media, and immunoprecipitated with anti-FLAG coupled beads to quantify its phosphorylation status. Expression of B1 and B2 promoted a significant increase in RTA phosphorylation relative to the control plasmid (Fig 6A). Furthermore, ^32^P orthophosphate labeling in MHV68-infected cells revealed that depletion of B2 RNA during infection reduced FLAG-RTA phosphorylation (Fig 6B).

Next, to determine whether this SINE-mediated phosphorylation change resulted in increased RTA activity, we examined the ability of RTA to transactivate several established RTA-responsive promoters in the presence or absence of B1 and B2 SINEs. Cells were co-transfected with RTA and luciferase reporter plasmids containing the RTA-responsive viral promoters from either RTA, ORF57, or M3 in the presence or absence of B1 and B2 SINE constructs (Fig 6C). Indeed, RTA mediated transcriptional activation of all three promoters was significantly enhanced upon co-expression of either B1 or B2 relative to the control plasmid. This ability of the SINE RNAs to enhance RTA activity was dependent on IKKβ phosphorylation of RTA, as an RTA mutant (RTA-TTS/A) [39] in which the IKKβ phosphorylation sites were mutated retained WT basal transcriptional activity but was unresponsive to the SINE RNAs (Fig 6C). Collectively, these data indicate that SINE RNA-mediated activation of IKKβ drives RTA phosphorylation, thereby increasing its activity on viral promoters and enhancing MHV68 gene expression.

## Discussion

**Fig 7:**
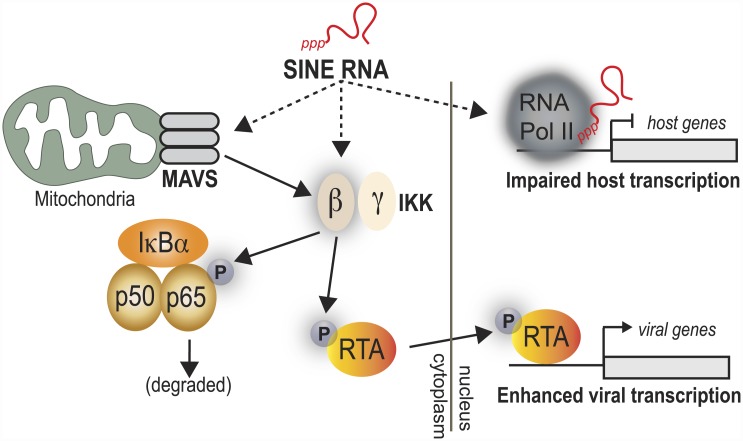
Model for SINE RNA enhancement of viral gene expression. SINE RNAs are robustly induced in response to MHV68 infection. Through both MAVS-dependent and independent mechanisms, they stimulate the activity of the IKKβ component of the NF-κB pathway. IKKβ is co-opted by the virus to promote phosphorylation of RelA/p65, blunting the NF-κB transcriptional response. Additionally, IKKβ phosphorylates RTA, thereby enhancing viral gene expression and replication.

Here, we demonstrate that MHV68-induced activation of SINE RNA serves to regulate viral and cellular gene expression through distinct mechanisms (Fig 7). SINE RNAs reside in the nucleus and in the cytoplasm, and participate in gene regulation in both compartments. Nuclear SINE RNAs appear absent from viral promoters but associate with specific cellular promoters that are repressed upon SINE activation, while SINE RNAs in the cytoplasm drive activation of the antiviral NF-κB signaling pathway. Though this pathway is normally detrimental to viral replication, MHV68 co-opts the IKKβ component of the NF-κB cascade to boost the activity of the viral lytic transactivator RTA, thereby enhancing viral gene expression and replication [39]. Thus, beyond direct regulation of cellular gene expression, induction of this class of ncRNAs may be linked to early immune-based sensing of infection, a process that has been hijacked by gammaherpesviruses to enhance viral gene expression.

Activation of SINE expression following MHV68 infection is a biphasic response with an initial phase arising as a result of either viral attachment or entry in to cells, and a second response that requires progression of the infection past entry, and includes immediate early and early viral gene expression. While it is possible that the initial burst of SINE expression observed following infection with U.V. inactivated virus is mediated by protein components of the tegument, we disfavor this scenario as we have been unable to identify an individual MHV68 gene whose expression is sufficient to induce SINE expression. This suggests that the mechanism by which MHV68 induces SINE expression is distinct from that of HSV-1 and Ad5, in which specific viral proteins have been implicated in promoting SINE induction. For instance, in the context of Ad5 infection, loss of E1a, E1b, or E4 ORFs 3 and 6 results in a significant decrease in SINE expression in infected cells [24]. Additionally, HSV-1 ICP27 has been demonstrated to enhance the activity of the RNA Pol III general transcription factor TFIIIC leading to increased SINE RNA expression [48]. In the case of MHV68, we believe a more likely model is one that focuses on the cellular stresses imposed on the cell during infection, including disruption of plasma membrane homeostasis during viral attachment and entry, and triggering of the innate immune response. This suggests that at least the initial burst of SINE transcription should occur regardless of whether infection progresses to latency or lytic replication. However, whether a latent infection results in sustained SINE activation similar to a lytic infection is currently unknown. As expression of SINE RNA potentiates a normally antiviral NF-κB response, the coupling of viral entry receptors and/or plasma membrane homeostasis to the production of immunogenic RNAs would constitute a means to rapidly prime the innate immune response for a potential pathogen exposure.

SINE induction may also occur as an early component of innate immune sensing. Indeed, many pathogen recognition receptors (PRRs) such as the toll like receptors (TLRs), which are present at both the cell surface and endosome, are engaged by herpesviruses [49]. In this regard, cell surface associated TLR2 has been implicated in detecting MHV68, likely recognizing viral glycoproteins [50]. Alternatively or perhaps concomitantly, the incoming viral DNA may be sensed by nucleic acid sensing PRRs, for which both endosomal TLR9 and the cytoplasmic cGAS-STING sensing pathways have been implicated [51–54]. As both stimulation of PRRs and exogenous overexpression of SINEs can mediate NF-κB, the induction of SINEs in response to PRR stimulation could establish a feed forward or signal amplification mechanism. In support of a model in which SINE RNAs are generated in response to PRR stimulation are the recent findings that lipopolysaccharide (LPS) stimulation of TLR4 induces a general activation of RNA Pol III transcription [55]. Though we do not detect a general increase in all RNA Pol III synthesized RNAs, its possible that MHV68 has evolved ways to manipulate the RNA Pol III transcriptional program to selectively drive synthesis of SINE RNAs, which enhance the MHV68 lifecycle. Interestingly, stimulation of TLRs 3,4,5, and 9 promote MHV68 reactivation from latently infected B cells [56].

SINE RNAs are present in both the cytoplasm and nucleus, and our and others’ data argue that they regulate gene expression via multiple mechanisms, some of which are linked to their location within the cell. For MHV68 it is the cytoplasmic fraction that is likely important for enhancing viral gene expression through the activation of the NF-κB pathway. Multiple studies have established that the NF-κB pathway is crucial for gammaherpesvirus latent infection [57], although reports have been more varied as to its roles in the viral lytic cycle. For instance, suppression of NF-κB signaling via expression of the IκBα super suppressor did not impair viral replication [43], whereas overexpression of the RelA/p65 subunit of the transcriptionally active NF-κB dimer inhibited the MHV68 lytic cycle [58]. However, these apparently inconsistent findings have recently been clarified by Feng and colleagues, who revealed that MHV68 activates the NF-κB pathway immediately following infection but that the downstream NF-κB transcriptional response is blunted as RelA/p65 is robustly targeted for degradation early in infection [39, 41]. Furthermore, optimal MHV68 gene expression and replication require the role of the IKKβ component of the NF-κB pathway [39, 41], which operates upstream of the IκBα super suppressor. IKKβ promotes phosphorylation of RelA/p65, thereby priming it for proteasomal degradation, as well as phosphorylation of MHV68 RTA, which enhances its transcriptional activating properties.

Interestingly, MHV68 activates IKKβ in both MAVS-dependent and independent ways. While some of the MAVS-dependent activation likely comes from MHV68-induced RIG-I deamidation [59], we establish that B2 SINEs are necessary for robust IKKβ activation, and that this also occurs through both MAVS-dependent and independent mechanisms. Though the RNA ‘sensor’ for SINE RNA is not known, the antiviral dsRNA binding protein PKR has been shown to bind to SINE RNA [34]. Furthermore, PKR is capable of directly associating with MAVS [60, 61]. Additionally, many other RNA sensors including RIG-I preferentially recognize many features present in SINE RNA, including 5’-triphosphate moieties and double stranded regions. However, we have not detected B2 RNAs in RNA immunoprecipitations of RIG-I, suggesting RIG-I may not be responsible for ‘sensing’ B2 RNAs. Future studies aimed at characterizing the composition of viral-induced SINE ribonucleoprotein complexes may provide insight here.

Many viruses, including the related gammaherpesvirus EBV, up regulate host RNA Pol III transcription [62]. In the case of EBV, the up regulation of RNA Pol III transcription is more general, although whether this includes SINE elements is unknown. Interestingly, it was recently shown that EBV infection stimulates RNA Pol III dependent expression of vault RNAs, which can also activate NF-κB and enhance viral establishment [63]. These results suggest that multiple RNA Pol III ncRNAs engage components of the cytoplasmic innate immune system, and their transcriptional induction in response to viral infection could be one of the early stress responses of the cell. Additionally, the production of abundant dsRNAs following reactivation of latent KSHV in iSLK cells was recently reported [64]. Whether the dsRNAs detected are human SINE RNAs is unknown but worthy of investigation.

SINE RNA expression also manipulates host gene expression. For example, during the heat shock response, global RNA Pol II transcription is down regulated. This is mediated in part through the direct binding of B2 RNAs to RNA Pol II, which prevent it from establishing contacts with the promoter during closed complex formation [26–28]. Our knockdown data coupled with our ChIRP-qPCR analyses reveal that B2 RNA-mediated transcriptional repression can similarly operate during viral infection, perhaps also through an interaction with RNA Pol II. At the moment we are unaware of the extent to which this occurs globally during infection. However, data from our laboratory suggest that it is unlikely that B2 RNAs globally repress transcription during MHV68 infection, as both WT virus and a viral mutant unable to restrict host gene expression induce B2 RNAs to a similar extent, yet widespread transcriptional repression is only observed during a WT infection [65]. In this regard, future studies will be important to determine the basis for promoter specificity.

SINE RNAs are also abundantly expressed early during embryogenesis within stem cells, a cell type in which dsRNA sensing is inefficient. The function of these ncRNAs within this context is unclear, though a fraction of SINE RNAs are processed into endosiRNAs [66, 67]. Interestingly, it has recently been reported that that efficient induction of induced pluripotent stem cells (iPSCs) requires activation of the dsRNA sensor TLR3 [68]. Whether continued low-level activation of the innate immune system is required for iPSC maintenance is unclear, but it is possible that SINE RNA expression provides chronic low-level innate immune stimulation to promote stem cell maintenance.

The use of viruses to perturb host systems, such as described here, presents a valuable platform to probe SINE ncRNA biology. SINE ncRNAs are a critical component of the gammaherpesvirus lifecycle, but they are also activated upon infection with multiple other human and murine viruses. Whether in other systems SINE RNAs serve as anti-viral signaling components, as well as if and how they are co-opted by the diversity of viral and non-viral pathogens remain exciting avenues for future research.

## Materials and Methods

### Cells, viruses, and infections

NIH3T3 (ATCC), NIH3T12 (ATCC), Vero, MEF, and WT and MAVS^-/-^ fibroblasts (kindly provided by Russell Vance, University of California Berkeley) were maintained in Dulbecco's modified Eagle medium (DMEM; Invitrogen) supplemented with 10% fetal bovine serum (FBS; Invitrogen). The green fluorescent protein (GFP)-expressing MHV68 bacterial artificial chromosome (BAC) has been described elsewhere [69]. BAC-derived MHV68 virus was produced by transfecting BAC DNA into NIH3T3 cells using SuperFect (Qiagen). Virus was then amplified in NIH 3T12 cells and titered by plaque assay on NIH3T3 cells. Before infecting mice, the loxP-flanked BAC vector sequence was removed by passaging the virus through Vero cells expressing Cre recombinase (kindly provided by Dr. Samuel Speck, Emory University).

### Nucleic acid isolation and measurement, and Luciferase assays

For analysis of gene expression by RT-qPCR, total or subcellular fractions of RNA were isolated with TRIzol (Invitrogen) in accordance with the manufacturer's instructions. cDNA was synthesized from 1 μg of RNA with random hexamers (Integrated DNA Technologies) and SuperScript II reverse transcriptase (Invitrogen). qPCR was performed using the DyNAmo ColorFlash SYBR green qPCR kit (Thermo Scientific) with appropriate primers.

For small RNA northern blot analysis total RNA was separated on 8% polyacrylamide–7M urea gels and electrotransferred at 4°C to Amersham Hybond-N+ membranes in 0.5X TBE buffer for 16h at 15V. Membranes were probed overnight using ^32^P-end labeled probes overnight at 55°C. Blots were washed three times in 0.1X SSC for 10 min each before exposed to phosphoimager screens overnight. For mRNA northern blot analysis total RNA was resolved on 1.2% agarose-formaldehyde gels and transferred to Hybond-N+ membranes by capillary action.

For primer extension, 15 μg of total RNA was incubated with 6 pmol ^32^P-labeled primer in 10 μl of Buffer A (250 mM KCl, 10 mM Tris, pH 7.5, and 1 mM EDTA) for 1 h at 55°C. Buffer B (40 μl) and 0.5 μl AMV reverse transcriptase (Promega) were added and incubated at 42°C for 1 h. Products were phenol-chloroform extracted, ethanol-precipitated, and resuspended in RNA loading dye before being resolved on 8% polyacrylamide–7M urea gels.

Nuclear run-on was performed as described previously with minor modifications [70]. Specifically, nuclei were isolated from two 10-cm plates of confluent mock- or MHV68 infected cells.

For Luciferase assays NIH3T3 cells were transfected in 6 well dishes with the indicated plasmids using TransIT-3T3 (Mirus Bio). 48 h post-transfection, lysates were prepared from approximately equal number of cells and luciferase activity was determined with the Promega luciferase assay system.

### Chromatin isolation by RNA purification (ChIRP)

ChIRP was performed as previously described with minor modifications [71]. Briefly, ~ 100 million NIH3T3 cells were infected with MHV68 at an MOI 5. 24 hpi cells were cross-linked with 1.1% formaldehyde for 15 min at room temperature. Crosslinking was then quenched with 0.125 M glycine for 5 min. Cells were rinsed again with PBS, scraped into Falcon tubes, and pelleted at 1000 g for 5 min. The cell pellet was resuspended in 3 mL nuclei lysis buffer (50 mM Tris-HCl [pH 7.0], 10 mM EDTA, 1% SDS, protease cocktail inhibitor [Roche], and RNAse inhibitor [Fermentas]) and rotated for 10 min. at 4°C. Cells were dounced 10 times with a B type pestle and separated in to three 1 mL aliquots for sonication. Sonication was performed using a Covaris focused sonicator. After sonication chromatin aliquots were combined and 9 mL of hybridization buffer (750 mM NaCl, 1% SDS, 50 mM Tris 7.0, 1 mM EDTA, 15% Formamide, protease inhibitor cocktail, and RNAse inhibitor) was added. 50 pmol of five separate 3’-TEG biotinylated probes was added to the dilute chromatin and rotated over night for 16 h. Streptavidin-magnetic C1 beads (Life Technologies) were washed three times in nuclei lysis buffer, blocked with 500 ng/μl yeast total RNA, and 1mg/ml BSA for 1 hr at room temperature, and washed three times again in nuclear lysis buffer before being resuspended in its original volume. One hundred microliters washed/blocked C1 beads were added to the chromatin mixture and rotated for an additional 4 h at 37°C. Beads:biotin-probes:RNA:chromatin adducts were captured by magnets (Invitrogen) and washed five times with 10 mL wash buffer (2× SSC, 0.5% SDS). After the last wash complexes were eluted by resuspending the beads in 500 μL G50 buffer (20 mM Tris-HCl, 300 mM NaCl, 2 mM EDTA, 0.2% SDS) plus 50 μg/mL Proteinase K (Fermentas) and incubating at 60°C for 1 h. The G50 buffer was separated from the beads, phenol chloroform extracted, and ethanol precipitated. RNA was analyzed by small RNA northern blotting and DNA was analyzed by qPCR, as described above.

### In vivo labeling/phosphorylation assay, cellular fractionation and western blotting

For in vivo kinase assays in the absence of infection, NIH3T3 cells were cotransfected with FLAG-RTA and either a B1 SINE, B2 SINE, or control expression plasmid using TransIT-3T3 (Mirus Bio). 24 h post-transfection media was replaced with phosphate‐free DMEM (Life Technologies) for 1 h and then incubated in the same medium containing [^32^P]-orthophosphate (0.5 mCi/ml final concentration, carrier free; PerkinElmer) for 6 h. After labeling cells were lysed in high salt RIPA buffer (20 mM Tris-HCl [pH 7.5], 500 mM NaCl, 1 mM EDTA, 1 mM EGTA, 1% NP-40, 1% sodium deoxycholate) containing a phosphatase inhibitor cocktail (Sigma) and immunoprecipitated using anti-FLAG (M2) magnetic beads (Sigma) overnight at 4°C. Beads were washed extensively with high salt RIPA buffer and then eluted with FLAG peptide (Sigma). The eluate was resolved by SDS–PAGE, transferred to PVDF membrane (Immobilon; Millipore) and visualized by autoradiography. Additionally western blot was performed as described below using anti-FLAG (M2, Sigma).

For in vivo labeling during MHV68 infection, NIH3T3 cells were transfected with FLAG-RTA as above. 24 h post-transfection cells were transfected with ASO’s using RNAiMAX as previously described [72]. 3 h post-ASO treatment the media was replaced with phosphate‐free DMEM (Life Technologies) for 1 h and then incubated in the same medium containing [^32^P]-orthophosphate (0.5 mCi/ml final concentration, carrier free; PerkinElmer) and infected with MHV68 at an MOI 5 for 6 h. Immunoprecipitations and downstream analysis was performed as described above.

Subcellular fractionation was performed using the REAP method with the minor modification of using one 10-cm plate for each fractionation condition [73].

For western blot analysis, cell lysates were prepared in NET-2 buffer (50 mM Tris-HCl [pH 7.6], 150 mM NaCl, 3 mM MgCl_2_, 10% glycerol, 0.5% Nonidet P-40), and protein concentrations were determined by Bradford assay. Equivalent quantities of each sample were fractionated by SDS-PAGE, transferred to a polyvinylidene difluoride membrane, and incubated with the appropriate antibodies. Western blot assays were developed with HRP-conjugated secondary antibodies and ECL reagents (Pierce).

### Growth curves and in vivo experiments

For multi-step growth curves, 1.5×10^5^ NIH3T3, WT or MAVS^-/-^ fibroblasts were infected with MHV68 at MOI of 0.05 and both supernatant and cells were harvested at 0, 1, 2, and 3 dpi and frozen. Samples were freeze-thawed once before titering by plaque assay on NIH3T3 cells.

Female C57BL/6J mice were obtained from The Jackson Laboratory (Bar Harbor, ME) and infected when 4–6 weeks old. Mice were anesthetized with isoflourane and inoculated intranasally with 5×10^4^ plaque forming units (pfu) in 20 μl DMEM (Invitrogen). Lungs were harvested 5 dpi and homogenized with a tissue homogenizer in 5 mL Trizol and RNA was isolated as described above.

### Ethics statement

This study was carried out in strict accordance with the recommendations in the Guide for the Care and Use of Laboratory Animals of the National Institutes of Health. The protocol was approved by the Committee on the Ethics of Animal Experiments of the University of California Berkeley (Permit Number: R292-0507). All animals were anesthetized prior to infection with isoflurane, and all efforts were made to minimize suffering.

## Supporting Information

S1 Fig Related to Fig 3: Inhibition of RNA Pol III transcription inhibits viral replication and gene expression. (**A**) NIH3T3 cells were pretreated with 40 μM ML-60218 for 6 h prior to infection with MHV68 at an MOI 5 for 24 h. Infectious virus was quantified by plaque assay on NIH3T3 cells. (**B**) RNA isolated 24 hpi was used to monitor the levels of viral and cellular RNAs by RT-qPCR.
(EPS)
